# The accuracy of general practitioner workforce projections

**DOI:** 10.1186/1478-4491-11-31

**Published:** 2013-07-16

**Authors:** Malou Van Greuningen, Ronald S Batenburg, Lud FJ Van der Velden

**Affiliations:** 1 NIVEL, Netherlands Institute for Health Services Research, Otterstraat 118-124, 3513 CR, Utrecht, The Netherlands; 2 Department of Information and Computing Sciences, Utrecht University, Princetonplein 5, 3584 CC, Utrecht, The Netherlands

**Keywords:** Health workforce, Projections, Evaluation, Accuracy

## Abstract

**Background:**

Health workforce projections are important instruments to prevent imbalances in the health workforce. For both the tenability and further development of these projections, it is important to evaluate the accuracy of workforce projections. In the Netherlands, health workforce projections have been done since 2000 to support health workforce planning. What is the accuracy of the techniques of these Dutch general practitioner workforce projections?

**Methods:**

We backtested the workforce projection model by comparing the ex-post projected number of general practitioners with the observed number of general practitioners between 1998 and 2011. Averages of historical data were used for all elements except for inflow in training. As the required training inflow is the key result of the workforce planning model, and has actually determined past adjustments of training inflow, the accuracy of the model was backtested using the observed training inflow and not an average of historical data to avoid the interference of past policy decisions. The accuracy of projections with different lengths of projection horizon and base period (on which the projections are based) was tested.

**Results:**

The workforce projection model underestimated the number of active Dutch general practitioners in most years. The mean absolute percentage errors range from 1.9% to 14.9%, with the projections being more accurate in more recent years. Furthermore, projections with a shorter projection horizon have a higher accuracy than those with a longer horizon. Unexpectedly, projections with a shorter base period have a higher accuracy than those with a longer base period.

**Conclusions:**

According to the results of the present study, forecasting the size of the future workforce did not become more difficult between 1998 and 2011, as we originally expected. Furthermore, the projections with a short projection horizon and a short base period are more accurate than projections with a longer projection horizon and base period. We can carefully conclude that health workforce projections can be made with data based on relatively short base periods, although detailed data are still required to monitor and evaluate the health workforce.

## Background

One of the major challenges in health-care systems worldwide is that of managing the health workforce to meet the demands of an accessible and effective health service. Shortages and imbalances of health-care personnel are a major concern of health policy-makers, professional bodies and patient organizations [1-5]. Health workforce planning is an important instrument to prevent shortages and oversupply within the health-care workforce [6-9]. An increasing number of countries apply different types of health workforce planning. Recently, Matrix Insight [10] conducted a study that provides an overview of health workforce planning in the European Union and shows a large variation across countries. Thirteen European countries, including the Netherlands, engage in model-based workforce planning, all of which use some form of supply-side projections.

Health workforce projections require accurate and comprehensive information and careful accounting of stocks and flows of human resources for health [10]. In most settings, the results and methods of workforce projections are not monitored and evaluated regularly and, consequently, it is difficult to assess whether workforce planning has been successful and projections are accurate. This implies that shortcomings and room for improvement are difficult to identify [10]. For the feasibility and further development of workforce projections in rapidly changing health systems, it is important to evaluate the accuracy of projections and their techniques [11]. The increasing dynamics of the health workforce – through mobility [12], reduction of working hours, the ageing workforce, increasing number of female physicians, changing division of labour – implies that projecting the future workforce could become more difficult [13-16].

### The accuracy of the Dutch simulation model

A simulation model had been developed in 2000 to support health workforce planning in the Netherlands. This model calculates the required number of health professionals in training to advise the Ministry of Health on the adjustment of the inflow numbers per year, to balance the supply and demand and to prevent a shortage or an oversupply of health professionals in the future [17-20].

**Figure 1:**
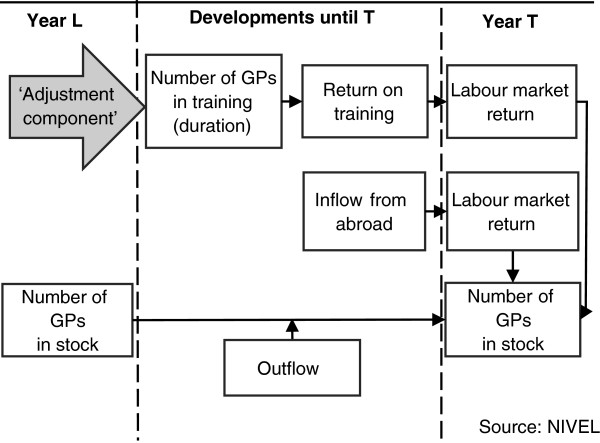
Supply side of the Dutch projection model for the health workforce.

Comparable with the techniques used for population projections, the Dutch workforce projection model is a cohort component model [21-23]. The components consist of inflow to or outflow from the active workforce. Figure 1 shows the supply side of the conceptual Dutch simulation model, of which the projection accuracy is studied in this article. The model is divided into three different stages that are related to the current situation (launch year), the developments between the current situation and the future (target year), and the situation in the target year.

The launch year is the year of the latest data used as a basis to make a projection and the target year is the projection year. Other terms used in this article are the projection horizon, which is the interval between launch year and target year, and the base period, which is the period of data the projection is based on (the interval between base year and launch year, with the base year being the year of the earliest data).

In the Netherlands, general practitioners (GPs) are of high importance as they provide primary health care 24 hours a day, 7 days a week and are the ‘gatekeepers’ of the health-care system [13]. Additionally, there is much data available about the Dutch GP, because the Netherlands Institute for Health Services Research (NIVEL) administers a GP database, which provides longitudinal information about all Dutch GPs regarding gender, age, position, moment of first-time accreditation, etcetera since 1975 [14,19,24,25].

The question of this article is: what is the accuracy of the current model for Dutch GP workforce projections? To answer this question, we will conduct a posteriori projections to backtest the current workforce projections and compare the projected ex-post number of GPs with the observed number of GPs. In practice, the Dutch GP workforce is projected with a base period of 15 years. Long-term data are used to prevent base data being influenced by fluctuations. Specifically, we will compare the accuracy of projections based on 15-year base periods and based on 5- or 10-year base periods to investigate if a shorter base period is as accurate as a 15-year base period.

There is no standard for workforce projection horizon lengths, but in European countries, a 10-year projection horizon is common [26,27]. In the Netherlands, it is common to make projections with horizons of 10 and 15 years, because of the relatively long period of physician training. Furthermore, it takes one or two whole years to adjust the inflow in training, because of the decision-making process [20]. The accuracy of different lengths of projection horizons is tested.

There is extensive literature available on the accuracy of population projections. In many of these studies the projection horizon and base period are addressed. Based on these studies, we expect that the accuracy of the GP workforce projections is influenced by the lengths of the projection horizon [11,22,28-30], base period [22,30-32] and the combination of the two [22,32]. The following expectations will be tested in this study:

1. The longer the projections, the lower the accuracy of the Dutch GP workforce projection model is.

2. The shorter the base period, the lower the accuracy of the Dutch GP workforce projection model is, because short base periods could be influenced by fluctuating data.

3. The accuracy of the Dutch GP workforce projection model will be highest when the lengths of the base period and the projection horizon are similar. Hypothesis 3 is not dependent on hypotheses 1 and 2.

## Methods

Backtesting (or hindcasting) is the process of evaluating a strategy, theory, or model by applying it to historical data. A key element of backtesting that differentiates it from other forms of historical testing is that backtesting calculates how a strategy would have performed if it had actually been applied in the past. This requires the backtest to replicate the conditions of the time in question in order to get an accurate result. In this article, the Dutch GP workforce projection model is backtested [33,34] by comparing a posteriori projections with the observed number of GPs in the target years. The projections of the GP workforce are made using the current version of the workforce simulation model and historical GP workforce data retrieved from the NIVEL GP database. The only way we can evaluate the current model is by using historical data to generate new projections. Original projections are not available to evaluate the performance of the simulation model.

All data and assumptions used in the projections are – depending on the length of the base period – based on 5-year averages from preceding periods (0 to 5 years, 0 to 10 years and 0 to 15 years back), except for the inflow in training. This inflow is not based on an average of historical data, but the observed inflow in training is used to test the accuracy of the modelling techniques. The reason for this is that the workforce simulation model actually has influenced the inflow in GP training in the past – as its results are taken into account by the Ministry and stakeholders in their decision about GP training inflow in the Netherlands [20]. Hence, the observed inflow in training is used in the a posteriori projections to exclude past interference of policy decisions with regard to training inflow. Using inflow projections made in the past would obviously blur the method of backtesting as applied in this study.

The equation that lies behind the conceptual projection model (Figure 1) is as follows:

$$\hat{n}GP_{T,X,Y}=\mathrm{nG}P_{T,X}-\hat{n}\mathrm{OU}T_{T,X,Y}+\hat{n}IN_{T,X,Y}+\epsilon_{T,X,Y}$$

nGP = number of GPs; nOUT = number of outflow; nIN = number of inflow; T = target year; X = projection horizon; Y = base period; ϵ = projection error.

The total estimated supply of GPs in the future $\left(\hat{n}GP_{T,X,Y}\right)$ is calculated using the GPs in stock in the launch year (*nGP*_*T*,*X*_), minus the estimated outflow $\left(\hat{n}\mathrm{OU}T_{T,X,Y}\right)$, plus the estimated inflow $\left(\hat{n}IN_{T,X,Y}\right)$ of GPs in the years between launch and target year (*T* − *X* → *T)*, based on a specific base period (*T* − *X* − *Y* → *T* − *Y)*. For example, to predict the number of GPs in 2011 (e.g. 12 000), the number of GPs in stock in 2006 is used (e.g. 10 000). The estimated outflow between 2006 and 2011 (e.g. 2 000) is subtracted from the 2006 GP number and the estimated inflow between 2006 and 2011 (e.g. 4 000) is added to the 2006 GP number to predict the 2011 number. The estimated outflow and inflow numbers are based on observed data between 2001 and 2006.

The estimated inflow $\left(\hat{n}IN_{T,X,Y}\right)$ is composed of several parts: the inflow from abroad and its labour market return, and the inflow from Dutch training and its return on training and labour market return. For example, the estimated inflow between 2006 and 2011 (e.g. 4 000) is calculated by multiplying the inflow from abroad between 2006 and 2011 (e.g. 250) with the labour market return of this inflow (e.g. 80%) and then add the inflow from Dutch training (e.g. 4 200) multiplied by the return on training (e.g. 95%) and its labour market return (e.g. 85%).

Several sources provide information for the projections. This is mainly the NIVEL GP database, which provides information about the GP stock [24]. Other sources are the training institutions and the Medical Accreditation Committee, which provide data for elements of the model, such as return on training [20].

The GP database is administered according to Dutch privacy legislation. The privacy regulation was approved by the Dutch Data Protection Authority. According to Dutch legislation, approval by a medical ethics committee was not required for this kind of data collection.

### Calculating the projection errors

The accuracy of the a posteriori GP workforce projections is backtested for three different projection horizons (X) and three different base periods (Y). By comparing the results of the projections with the observed number of GPs (for the target years 1998 to 2011), the mean absolute percentage errors (MAPE) are calculated. The MAPE is a summarizing measure to express the error during a certain period of time and ignores the direction of error. It has been used frequently in evaluations of population forecast accuracy [23,35,36].

It is calculated for three projection horizons (MAPE_X_), three base periods (MAPE_Y_) and all combinations (MAPE_X,Y_). The equations are:

$$\begin{array}{l} \mathrm{MAP}E_{X,Y}=\frac{\sum^{T}\left|\left({\mathrm{projection}}_{T,X,Y}-,{\text{observation}}_{T}\right)\right|\cdot 100\%}{\sum^{T}{\text{observation}}_{T}}\\ \;=\frac{\sum^{T}\epsilon_{T,X,Y}}{\sum^{T}\mathrm{nG}P_{T}} \end{array}$$

$$\begin{array}{l} \mathrm{MAP}E_{X}=\frac{\sum^{Y}\sum^{T}\left|\left({\mathrm{projection}}_{T,X,Y}-{\mathrm{observation}}_{T}\right)\right|\cdot 100\%}{\sum^{T}{\mathrm{observation}}_{T}}\\ \;=\frac{\sum^{Y}\sum^{T}\epsilon_{T,X,Y}}{\sum^{T}\mathrm{nG}P_{T}} \end{array}$$

$$\begin{array}{l} \mathrm{MAP}E_{Y}=\frac{\sum^{X}\sum^{T}\left|\left({\mathrm{projection}}_{T,X,Y}-{\mathrm{observation}}_{T}\right)\right|\cdot 100\%}{\sum^{T}{\mathrm{observation}}_{T}}\\ \;=\frac{\sum^{X}\sum^{T}\epsilon_{T,X,Y}}{\sum^{T}\mathrm{nG}P_{T}} \end{array}$$

nGP = number of GPs; T = target year; X = projection horizon; Y = base period; ϵ = projection error.

The first target year, 1998, is determined by the first year of available data, 1968, and the sum of 15 years of base data and a 15-year horizon.

Table 1 defines the years and time periods on which observations are based and which are used to calculate the projection accuracy. The number of GPs is projected for every target year between 1998 and 2011 for a 5-, 10- and 15-year projection horizon. These projections are based on GP stock data of 5, 10 or 15 years earlier (for each launch year, 1983 to 2006) and on base periods of 5, 10 and 15 years (data between 1968 and 2001). Table 1 also depicts the projection accuracy for each of these calculations, which are further discussed in the results section.

**Table 1:** Years and time periods on which observations are based and which are used to calculate the projections’ accuracy

		**5-year base period**	**10-year base period**	**15-year base period**	**MAPE**_**x**_
**5-year projection horizon**	Base years	1988 - 2001	1983 - 1996	1978 - 1991	
	Base periods	1988 → 1993 –	1983 → 1993 –	1978 → 1993 –	
		2001 → 2006	1996 → 2006	1991 → 2006	
	Launch years	1993 - 2006	1993 - 2006	1993 - 2006	
	Projection horizons	1993 → 1998 –	1993 → 1998 –	1993 → 1998 –	
		2006 → 2011	2006 → 2011	2006 → 2011	
	Target years	1998 - 2011	1998 - 2011	1998 - 2011	
	MAPE_x,y_	1.9 (14 tests)	3.0 (14 tests)	4.2 (14 tests)	3.0 (42 tests)
**10-year projection horizon**	Base years	1983 -1996	1978 - 1991	1973 - 1986	
	Base periods	1983 → 1988 –	1978 → 1988 –	1973 → 1988 –	
		1996 → 2001	1991 → 2001	1986 → 2001	
	Launch years	1988 - 2001	1988 - 2001	1988 - 2001	
	Projection horizons	1988 → 1998 –	1988 → 1998 –	1988 → 1998 –	
		2001 → 2011	2001 → 2011	2001 → 2011	
	Target years	1998 - 2011	1998 - 2011	1998 – 2011	
	MAPE_x,y_	4.9 (14 tests)	7.0 (14 tests)	9.1 (14 tests)	7.0 (42 tests)
**15-year projection horizon**	Base years	1978 - 1991	1973 - 1986	1968 – 1981	
	Base periods	1978 → 1983 –	1973 → 1983 –	1968 → 1983 –	
		1991 → 1996	1986 → 1996	1981 → 1996	
	Launch years	1983 - 1996	1983 - 1996	1983 - 1996	
	Projection horizons	1983 → 1998 –	1983 → 1998 –	1983 → 1998 –	
		1996 → 2011	1996 → 2011	1996 → 2011	
	Target years	1998 - 2011	1998 - 2011	1998 - 2011	
	MAPE_x,y_	9.0 (14 tests)	12.4 (14 tests)	14.9 (14 tests)	12.1 (42 tests)
	MAPE_y_	5.3 (42 tests)	7.5 (42 tests)	9.4 (42 tests)	

Base year = earliest data year; base period = interval between base year and launch year; launch year = most recent data year; projection horizon = interval between launch year and target year; target year = projection years; number of accuracy tests = units of comparison between observed and projected number of GPs; MAPE = mean absolute percentage error. Example: to project the number of GPs for the target year 2011 with a projection horizon of five years, the projection is based on GP stock data from 2006 (launch year). The flow data used in the projection is based on data from 2001 to 2006 (five-year base period). 2001 is the base year.

### Analyses

Analyses were made using STATA 12 software. The first and second hypotheses were tested using the Kruskal-Wallis equality-of-populations rank test and the two-sample Wilcoxon rank-sum (Mann–Whitney) test. The first test was used for testing the difference between the percentage errors of three different horizon lengths (first hypothesis) and three different base period lengths (second hypothesis). The second test was used to test which of the three horizon and three base period lengths differ significantly. The third hypothesis was tested using the two-sample Wilcoxon rank-sum (Mann–Whitney) test. To test this hypothesis, the difference between, on the one hand, percentage errors of projections with similar horizon and base period lengths and, on the other hand, the percentage errors of projections with different horizon and base period lengths were tested.

## Results

The MAPEs that resulted from the analyses range from 1.9% to 14.9%. This means that, on an average of 8 801 GPs in the period 1998 to 2011, the projection error equals 167 to 1311 GPs. These numbers show a large range and are equal to one third to 2.5 times the size of the yearly inflow in GP training (almost 500 persons on average started the training every year between 1998 and 2011).

**Figure 2:**
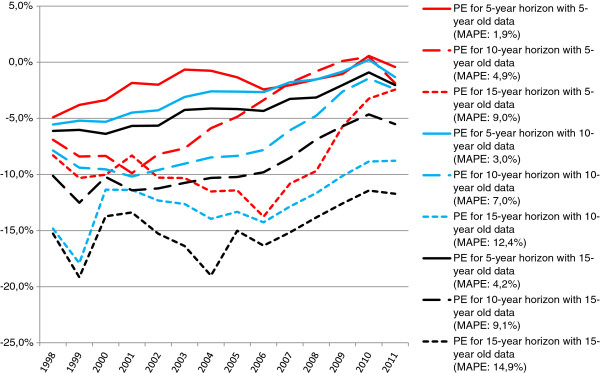
Accuracy (percentage error) of the Dutch projection model, by projection horizon length and base period length.

Figure 2 depicts the accuracy of three projection horizon lengths, each based on three base period lengths, for every year between 1998 and 2011. It shows that the number of GPs was underestimated in most years. Overall, the error of GP projections seems to be smaller in more recent years.

### Accuracy by length of projection horizon

To test the first hypothesis (the longer the projections, the lower the accuracy) the MAPE_X_ of projections with 5-, 10- and 15-year horizons are compared and the differences were tested. Table 1 shows that projections with a 5-year horizon have a higher accuracy than projections with a 10-year horizon, which subsequently have a higher accuracy than projections with a 15-year horizon.

The differences were significant according to the Kruskall-Wallis test (χ^2^ = 75.669; *P* = 0.0001). According to the two-sample tests (Wilcoxon), projections with 5- and 10-year horizons are different (z = 5.122; *P* = 0.0000), as well as projections with 10- and 15-year horizons (z = 5.896; *P* = 0.0000) and 5- and 15-year horizons (z = 7.497; *P* = 0.0000). Consequently, hypothesis 1 is confirmed. This is in accordance with earlier research [11,22,28-30]. In the present study, projections with a 5-year horizon are twice as accurate as projections with a 10-year horizon and four times as accurate as projections with a 15-year horizon.

### Accuracy by base period length

The second hypothesis (the longer the base period, the higher the accuracy) is tested by comparing the MAPE_Y_ of projections based on 5-, 10- and 15-year base periods (0 to 5 years, 0 to 10 years and 0 to 15 years before the launch year). Table 1 shows that projections with a 5-year base period have a higher accuracy than projections with a 10-year base period, which subsequently have a higher accuracy than projections with a 15-year base period. The differences were significant according to the Kruskall-Wallis test (χ^2^ = 15.826; *P* = 0.0004). According to the two-sample tests (Wilcoxon), projections with 5- and 10-year base periods are different (z = 2.246; *P* = 0.0247), as well as projections with 5- and 15-year base periods (z = 3.865; *P* = 0.0001). Projections with 10- and 15-year base periods are not different (z = 1.923; *P* = 0.0544). In conclusion, hypothesis 2 is not confirmed, because projections with a shorter base period are not less accurate.

### Accuracy by similarity of projection horizon length and base period length

The MAPE_X,Y_ of projections with three horizons based on 5-, 10- and 15-year base periods are compared to test the third hypothesis (the accuracy will be highest when the lengths of the base period and the projection horizon are similar). Table 1 shows that for every projection length, the projections with a 5-year base period have a higher accuracy than projections based on a 10-year period, which subsequently have a higher accuracy than projections based on a 15-year period. The differences between the errors of two groups were tested: projections with similar horizon and base period lengths and projections with different horizon and base period lengths. According to the two-sample tests (Wilcoxon), the errors of the two groups are not different (z = 0.391; *P* = 0.6960). Consequently, the accuracy is not highest when projection horizon length and base period length are similar and the third hypothesis is not confirmed.

## Discussion

The goal of this article was to evaluate the accuracy of the techniques of Dutch GP workforce projections by backtesting projections and comparing the a posteriori projections with the observed number of GPs in 1998 to 2011. Another goal was to test three hypotheses about the accuracy of different projection horizon and base period lengths.

According to the results of the present study, the projections with a short projection horizon and a short base period are more accurate than projections with a longer horizon and base period.

The Dutch health workforce projections usually have projection horizons of 10 and 15 years. According to the results, projections with a 5-year horizon are however the most accurate. This is in accordance with the results of studies regarding the accuracy of population projections [11,22,28-30]. Large errors in supply projections could cause an imbalance between supply and demand, and as a result major adjustments in training inflow would be needed. To minimize the errors in projections with a longer horizon, it is recommendable to monitor the workforce continuously and to execute projections frequently. In practice, it is not feasible to execute projections with a shorter projection horizon, because there would only be a short period to match supply and demand. Dramatic fluctuations in yearly training inflow would be needed to reach a balance between supply and demand. It is undesirable to adjust the inflow number in training by large numbers each year, because this would be practically impossible for training institutions, for example.

Dutch GP workforce projections that are carried out to advise the government are based on a 15-year base period. According to the results of the present study, projections with a 5-year base period are more accurate than those with a 10- or 15-year period. Consequently, a base period of 5 or 10 years also seems extensive enough to make reliable projections. It seems that the GP workforce of today is different from the past GP workforce and, therefore, we can conclude that base periods containing not only recent data but also older data are less representative for GPs in the target year. According to projections based on base periods including older data, we expected the GPs to leave the workforce at an earlier age than was observed. Current GPs stay in the workforce longer.

We can thus carefully conclude that health workforce projections can be made with data based on relatively short periods and less data, although detailed data are required to monitor and evaluate the health workforce [37].

The accuracy of the projections varies per year and there seems to be a trend towards more accurate projections in more recent years for all base period lengths. Hence, forecasting the size of the future workforce did not become more difficult between 1998 and 2011, as we originally expected [13-15]. This trend could be explained by two things. First, it seems that the GP workforce of 1980 to 1990 is less similar to the workforce of 1990 to 2000 than the workforce of 1990 to 2000 is similar compared to the workforce of 2000 to 2010. In other words, the GP workforce changed more extensively between 1980 and 1990 and 1990 and 2000 than between 1990 and 2000 and 2000 and 2010. Second, the Dutch GP workforce has become larger. Data based on a larger base population size have more stable averages than data based on smaller populations.

The errors of the Dutch GP workforce projections range from 1.9% to 14.9%. This is a large range, which illustrates the importance of doing projections with different projection horizon lengths and base period lengths.

The projection errors are mainly caused by bias and not by variance [38]. The variance is low, because data of all Dutch GPs is used to make projections. The projection error is mostly bias, caused by differences between the past GP workforce and the current and future GP workforce.

From a data availability perspective, it may be possible that there is significant scope for more countries to engage in model-based health workforce planning than is currently the case, and for countries already engaging in such planning to extend the reach of their current models, which was also concluded from the Matrix Insight report [10].

However, the successful application of a model similar to the Dutch workforce projection model is dependent on the health workforce planning system of a country. The output of the Dutch projection model is the required inflow in specialized training per year to balance the supply and demand for health professionals in the future [17-20]. Hence, the height of inflow in specialized training is the ‘adjustment component’ of the Dutch health workforce. In other health workforce planning systems, other parts of the planning system are possibly used as the ‘adjustment component’, such as postponing retirement or increasing the return on training (Figure 1). In Belgium, for example, the inflow in initial medical training (not specialized training) is the ‘adjustment component’ [39]. Future research is needed to investigate which type of health workforce planning fits with which type of health-care system [40].

### Limitations

This study has several limitations. First, in the present study, we backtested the current GP workforce projection methods a posteriori. There are other methods to analyse the accuracy of workforce projections, which we did not use. For example, we did not evaluate the current projections by comparing the results of GP workforce projections that were done in the past, with the actual observations. This second method seems simpler, but with this method we would not evaluate the current model, but older versions of it. The only way we can evaluate the current model, is by using old data to generate new projections. This is because future numbers are not yet known.

Second, the present study was limited to testing one health-care profession in the Netherlands: general practitioners. In practice, the model is used for all types of medical and allied health professionals, as the model is designed as ‘one size fits all’. This implies that the backtesting of projections is possible for all types of health professionals. However, for most of them there is less data available and therefore it is more difficult to backtest.

Third, the accuracy of the demand side of the Dutch health workforce simulation model was not tested, because of a lack of data. However, this should be a topic of future research on the accuracy of the Dutch health workforce planning system [18].

Fourth, testing the accuracy of workforce projections can be done disaggregated by several factors, such as gender, region, cohort or type of GP. Although this would have been an interesting exercise, we limited this study to the total supply of GPs. It would be an interesting case for future research.

## Conclusions

According to the results of the present study, forecasting the size of the future workforce did not become more difficult between 1998 and 2011, as we originally expected. Furthermore, the projections with a short projection horizon and a short base period are more accurate than projections with a longer projection horizon and base period. We can carefully conclude that health workforce projections can be made with data based on relatively short base periods, although detailed data are still required to monitor and evaluate the health workforce.

## Abbreviations

GP: General practitioner; MAPE: Mean absolute percentage error.

## Competing interests

The authors declare that they have no competing interests.

## Authors' contributions

MVG drafted and revised the manuscript. LVDV is one of the designers of the original model. MVG and LVDV analysed the data and calculated the accuracy of the projections. RB and LVDV helped draft and revise the manuscript. All authors read and approved the final manuscript.

## References

[B1] Askildsen JE Baltagi BH Holmas TH Wage policy in the health care sector: a panel data analysis of nurses' labour supply Health Econ 2003 12 705 719 10.1002/hec.836 12950091

[B2] Commission of the European Communities Green paper on European Workforce for health 2008 Brussels: Commission of the European Communities COM 725

[B3] Correia I Veiga P Geographic distribution of physicians in Portugal The Journal of Health Economics 2010 11 383 393 10.1007/s10198-009-0208-8 20012127

[B4] OECD The looming crisis in the health workforce 2008 Paris: OECD Health Policy Studies, OECD Publishing

[B5] Simoens S Villeneuve M Hurst J Tackling nurse shortages in OECD countries 2005 Paris: OECD

[B6] Bloom BS Health manpower planning Health Serv Res 1980 15 67 68

[B7] Dreesch N An approach to estimating human resource requirements to achieve the Millennium Development Goals Health Policy Plan 2005 20 267 276 10.1093/heapol/czi036 16076934

[B8] Maynard A Walker A The physician workforce in the United Kingdom: issues, prospects and policies 1997 London: Nuffield Trust

[B9] Yett DE Drabek L Intriligator MD Kimbell LJ Health manpower planning Health Serv Res 1972 7 134 147 5044700 PMC1067403

[B10] Matrix Insight EU level collaboration on forecasting health workforce needs, workforce planning and health workforce trends. A feasibility study 2012 European Commission: Brussels

[B11] O'Brien-Pallas L Birch S Baumann A Tomblin Murphy G Integrating workforce planning, human resources, and service planning Human Resources for Health Development Journal 2001 5 2 16

[B12] Wismar M Health professional mobility and health systems: evidence from 17 European countries 2011 Copenhagen: WHO

[B13] Schäfer W The Netherlands: health systems review 2010 Copenhagen: World Health Organization

[B14] Van den Berg M Workload in general practice [PhD thesis] 2010 Amsterdam: GVO drukkers & vormgevers B.V. | Ponsen & Looijen

[B15] Dussault G Franceschini MC Not enough there, too many here: understanding geographical imbalances in the distribution of the health workforce Human Resources for Health 2006 4 12 10.1186/1478-4491-4-12 16729892 PMC1481612

[B16] Olsen KR Organisational determinants of production and efficiency in general practice: a population-based study Eur J Health Econ 2013 14 2 267 76 10.1007/s10198-011-0368-1 22143360

[B17] Advisory Committee on Medical Manpower Planning The 2010 recommendations for medical specialist training in medical, dental, clinical technological and related educational as well as further training areas 2011 Utrecht: Advisory Committee on Medical Manpower Planning

[B18] Smits M Slenter V Geurts J Pucihar A Improving manpower planning in health care Proceedings of the 23rd Bled eConference ‘eTrust: Implications for the individual, enterprises and society’ 2010 Bled: Slovenia 144 154

[B19] Van der Velden LFJ Hingstman L Westert GP, Jabaaij L, Schellevis FG The supply of general practitioners in the Netherlands Morbidity, performance and quality in primary care: Dutch general practice on stage 2006 Oxford: Radcliffe Publishing 257 264

[B20] Van Greuningen M Batenburg RS Van der Velden LFJ Ten years of health workforce planning in the Netherlands: a tentative evaluation of GP planning as an example Human Resources for Health 2012 10 21 10.1186/1478-4491-10-21 22888974 PMC3465184

[B21] De Beer J Transparency in population forecasting. Methods for fitting and projecting fertility, mortality and migration, [PhD thesis] 2011 Amsterdam: Amsterdam University Press

[B22] Rayer S Population forecast errors. A primer for planners Journal of Planning Education and Research 2008 27 417 430 10.1177/0739456X07313925

[B23] Smith S Sincich T Evaluating the forecast accuracy and bias of alternative projections for states International Journal of Forecasting 1992 8 495 508 10.1016/0169-2070(92)90060-M 12157868

[B24] NIVEL databases of health professionals(http://www.nivel.nl/beroepen-in-de-gezondheidszorg). Date of access: September 25^th^ 2012

[B25] van Dijk CE Changing the GP payment system: do financial incentives matter? [PhD thesis] 2012 Utrecht: LABOR Grafimedia BV

[B26] Dussault G Buchan J Sermeus W Padaiga Z Investing in Europe's health workforce of tomorrow: scope for innovation and collaboration. Assessing future health workforce needs 2010 Geneva: WHO

[B27] Sermeus W Bruyneel L Investing in Europe's health workforce of tomorrow: scope for innovation and collaboration. Summary report of the three Policy Dialogues 2010 Leuven: Catholic University Leuven

[B28] Shapiro JR Modeling the supply chain 2001 Pacific Grove, CA, USA: Duxbury

[B29] Evaluating population projections. The importance of accurate forecasting 2007 New York: Esri

[B30] Ahlburg DA Lutz W Lutz W, Vaupel JW, Ahlburg DA Introduction: the need to rethink approaches to population forecasts Frontiers of population forecasting. A supplement to Vol. 24 1998 New York: The Population Council 1 14

[B31] Smith S Sincich T On the relationship between length of base period and population forecast errors J Am Stat Assoc 1990 85 367 375 10.1080/01621459.1990.10476209 12155386

[B32] Alho J Spencer BD The practical specification of the expected error in population forecasts Journal of Official Statistics 1997 13 203 225

[B33] Dowd K Backtesting stochastic mortality models: an ex-post evaluation of multi-period-ahead density forecasts North American Actuarial Journal 2010 14 281 298 10.1080/10920277.2010.10597592

[B34] Dowd K Evaluating the goodness of fit of stochastic mortality models Insurance Mathematics and Economics 2010 47 255 265 10.1016/j.insmatheco.2010.06.006

[B35] Ahlburg D A commentary on error measures: error measures and choice of a forecast method International Journal of Forecasting 1992 8 99 111 10.1016/0169-2070(92)90010-7

[B36] Keilman N How accurate are the United Nations world population projections? Population and Development Review 1999 24 15 41

[B37] Diallo K Zurn P Gupta N Dal Poz M Monitoring and evaluation of human resources for health: an international perspective Human Resources for Health 2003 1 3 10.1186/1478-4491-1-3 12904252 PMC179874

[B38] Hastie T Tibshirani R Friedman J The elements of statistical learning. Data mining, inference, and prediction, Springer Series in Statistics 2008 New York: Springer

[B39] Artoisenet C Deliege D Medical workforce in Belgium: assessment of future supply and requirements Louvain Med 2006 125 4 21

[B40] Kuhlmann E Batenburg R Groenewegen PP Larsen C Bringing a European perspective to the health human resources debate: a scoping study Health Policy 2013 110 6 13 10.1016/j.healthpol.2012.11.002 23200603

